# Supplementary material for: EasyEyes: Crowded Dynamic Fixation for Online Psychophysics
Source: bioRxiv. 2025 Apr 21:2025.02.26.640403. Originally published 2025 Mar 2. Preprint. [Version 2] doi: 10.1101/2025.02.26.640403 (PMC11888485; doi:10.1101/2025.02.26.640403)
Supplement: Supplement 1 [file NIHPP2025.02.26.640403v2-supplement-1.pdf]

bioRxiv preprint doi: https://doi.org/10.1101/2025.02.26.640403; this version posted April 21, 2025. The copyright holder for this preprint (which was not certified by peer review) is the author/funder, who has granted bioRxiv a license to display the preprint in perpetuity. It is made available under aCC-BY 4.0 International license.

Supplementary figures

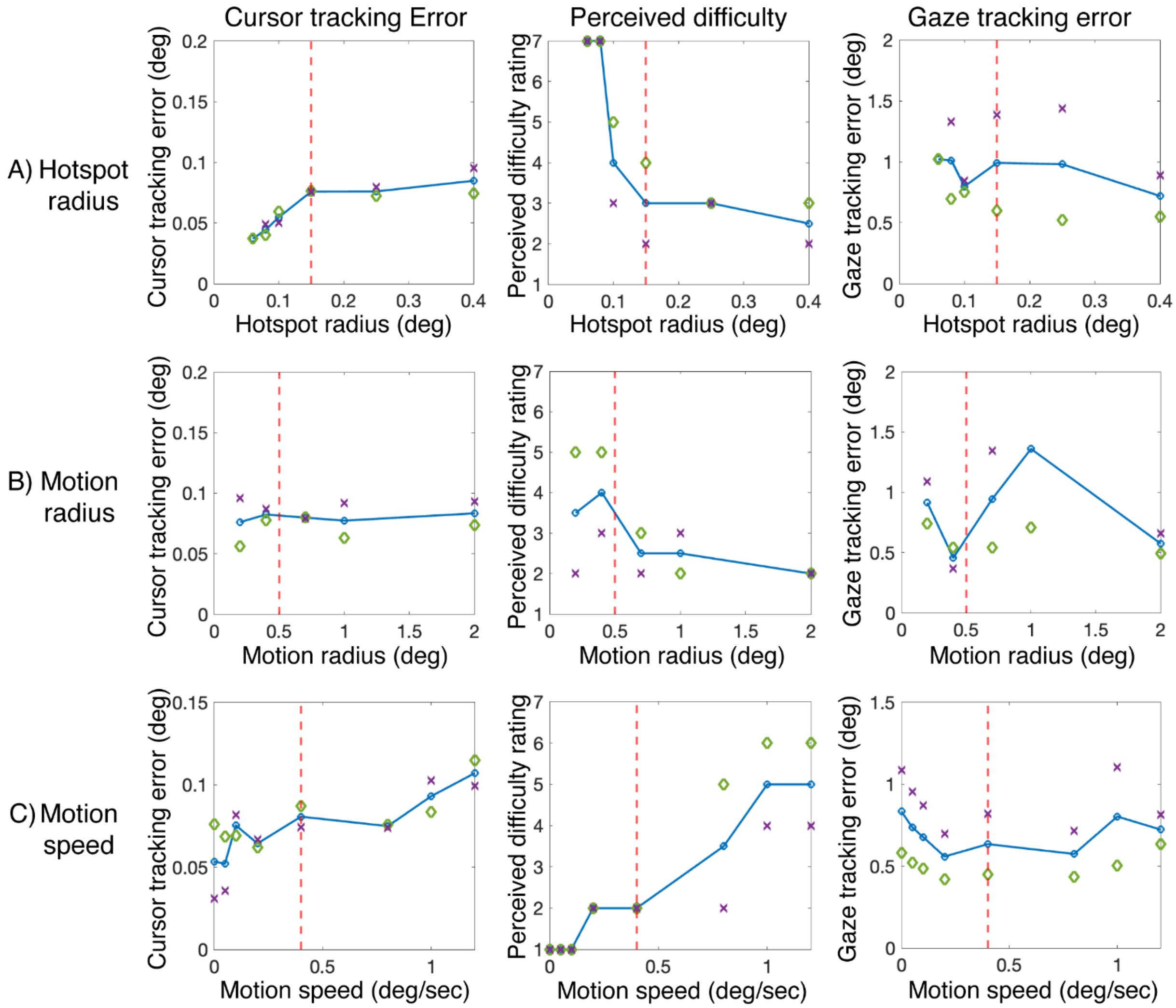

Supplement 1. Exploratory mapping of crosshair parameters, for adults. Vertical red dashed lines indicate the parameter values used in this paper. A) Hotspot radius: A radius smaller than 0.15 deg is too challenging, but a hotspot radius of 0.15 deg is moderately challenging and keeps the cursor and gaze tracking errors low; B) Motion radius: A motion radius below 0.4 deg is too challenging, and a radius larger than 0.4 deg produces too much gaze tracking error; C) Motion speed: Speeds below 0.2 deg/sec are too easy, and speeds much above 0.4 deg/sec are too challenging. *(compare_ee_el_wTimeouts.m)*
