# Supplementary material for: EasyEyes: Crowded Dynamic Fixation for Online Psychophysics
Source: bioRxiv. 2025 Mar 2:2025.02.26.640403. Preprint. [Version 1] doi: 10.1101/2025.02.26.640403 (PMC11888485; doi:10.1101/2025.02.26.640403)
Supplement: Supplementary file 1 [file NIHPP2025.02.26.640403V1-supplement-1.pdf]

bioRxiv preprint doi: https://doi.org/10.1101/2025.02.26.640403; this version posted March 2, 2025. The copyright holder for this preprint (which was not certified by peer review) is the author/funder, who has granted bioRxiv a license to display the preprint in perpetuity. It is made available under aCC-BY 4.0 International license.

Supplementary Figures

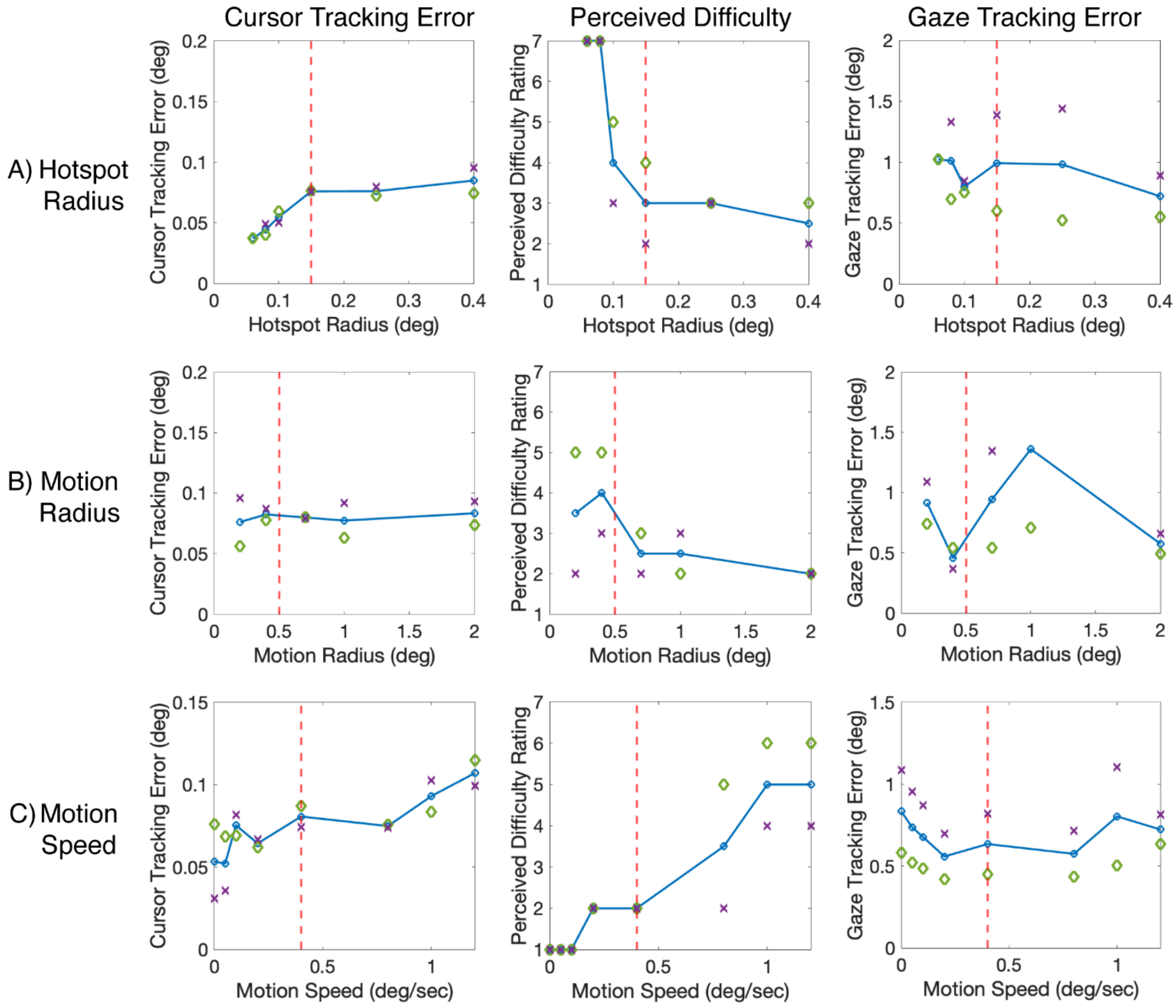

Supplement 1. Exploratory work for mapping out crosshair parameters. Red dashed lines indicate the parameters that were used in this paper. A)Hotspot radius: hotspot radii smaller than 0.15 deg are too challenging, but a hotspot radius of 0.15 deg is moderately challenging while keeping the tracking and gaze errors low; B) Motion Radius: Motion radius of 0.4 deg result in a moderate perceived difficulty and the lowest gaze error; C) Motion Speed: motion speeds lower than 0.4 deg/sec are too easy, while those larger than 0.4 deg/sec is too challenging. *(compare_ee_el_wTimeouts.m)*
